# Towards personal health care with model-guided medicine: long-term PPPM-related strategies and realisation opportunities within ‘Horizon 2020’

**DOI:** 10.1186/1878-5085-5-8

**Published:** 2014-05-30

**Authors:** Heinz U Lemke, Olga Golubnitschaja

**Affiliations:** 1 International Foundation for Computer Assisted Radiology and Surgery, 79790 Küssaberg, Germany; 2 European Association for Predictive, Preventive and Personalised Medicine, 1150 Brussels, Belgium; 3 Radiological Clinic, University of Bonn, Sigmund-Freud-Str. 25, 53105 Bonn, Germany

**Keywords:** Predictive preventive personalised medicine, Healthcare, ICT, Model-guided medicine, Evidence-based medicine, Medical information and model management system, Patient-specific model, Horizon 2020

## Abstract

At the international EPMA Summit carried out in the EU Parliament (September 2013), the main challenges in Predictive, Preventive and Personalised Medicine have been discussed and strategies outlined in order to implement scientific and technological innovation in medicine and healthcare utilising new strategic programmes such as ‘Horizon 2020’.

The joint EPMA (European Association for Predictive, Preventive and Personalised Medicine) / IFCARS (International Foundation for Computer Assisted Radiology and Surgery) paper emphasises the consolidate position of the leading experts who are aware of the great responsibility of being on a forefront of predictive, preventive and personalised medicine. Both societies consider long-term international partnerships and multidisciplinary projects to create PPPM relevant innovation in science, technological tools and practical implementation in healthcare. Personalisation in healthcare urgently needs innovation in design of PPPM-related medical services, new products, research, education, didactic materials, propagation of targeted prevention in the society and treatments tailored to the person. For the paradigm shift from delayed reactive to predictive, preventive and personalised medicine, a new culture should be created in communication between individual professional domains, between doctor and patient, as well as in communication with individual social (sub)groups and patient cohorts. This is a long-term mission in personalised healthcare with the whole spectrum of instruments available and to be created in the field.

## Introduction

At the international EPMA Summit carried out in the EU Parliament (September 2013), the main challenges have been emphasised and strategies outlined in order to implement scientific and technological innovation in medicine and healthcare as follows:

– Predictive, preventive and personalised medicine (PPPM) is the emerging field considered as the medicine of the future.

– PPPM is the patient-centred approach meeting healthcare challenges, running treatments efficiently and keeping costs of medical services under control.

– PPPM objectives promote innovation in science, technologies, education, healthcare, economical and social aspects of the societies in Europe and worldwide.

– Successful PPPM implementation needs unprecedented level of collaboration amongst all stakeholders, long-term multidisciplinary professional partnerships including public-private ones, robust juristic platform and smart political regulations.

– Gathering funding instruments focused on integration of different PPPM-related branches is essential to be introduced by new strategic programmes such as ‘Horizon 2020’.

At the EPMA Summit in the EU Parliament, Henri Malosse, President of the ‘European Economic and Social Committee’, EU, emphasised:

‘…we need a permanent information stream between healthcare providers and policy-makers to perform adequate political regulations and creation of new guidelines to advance healthcare-related science, technologies and implementation in daily medical practice. This innovation is provided by highly promising approach of Predictive, Preventive and Personalised Medicine. We are confident of the excellence of the initiatives of the European Association for Predictive, Preventive and Personalised Medicine (EPMA, Brussels) effectively promoting a paradigm shift from less effective delayed treatments to predictive, preventive and more personalised measures in healthcare as the medicine of the future. This innovation conducted by EPMA initiatives should be extensively supported.’

New Programme ‘Horizon 2020’ considers ‘integrated approach to address key research challenges’, namely:

– breaking barriers and speaking the same language

– ‘cross-disciplinarity’, education and training

– generating knowledge and developing the right tools

– standards, clinical bioinformatics and adaptation of tools

– translating knowledge to medical applications

– disease taxonomy, biomarker validation and clinical trials

– understanding the value and economic aspects

– healthcare pilots, HTA, comparative effectiveness, etc.

## Predictive, preventive and personalised medicine as the medicine of the future

Predictive, preventive and personalised medicine (PPPM) is the new integrative concept in healthcare sector that enables to predict individual predisposition before onset of the disease, to provide targeted preventive measures and create personalised treatment algorithms tailored to the person. The innovative PPPM is emerging as the focal point of efforts in healthcare aimed at curbing the prevalence of both communicable and non-communicable diseases such as diabetes, cardiovascular diseases, chronic respiratory diseases, cancer and dental pathologies. The expected outcomes are conducive to more effective population screening, prevention early in childhood, identification of persons at risk, stratification of patients for the optimal therapy planning, prediction and reduction of adverse drug-drug or drug-disease interactions relying on emerging technologies, such as pharmacogenetics, pathology-specific molecular patters, sub/cellular imaging, disease modelling and individual patient profiles. Integrative approach by PPPM is considered as the medicine of the future. The cost-effective management of diseases and the critical role of PPPM in the modernisation of healthcare have been acknowledged as priorities by global and regional organisations and health-related institutions such as the Organisation of United Nations, the European Union and the National Institutes of Health [1].

## PPPM requires effective professional consolidation and new culture of multi-disciplinary communication: barriers, obstacles and solutions

Through the support of the Alexander von Humboldt Foundation (2012–2013), the European Association for Predictive, Preventive and Personalised Medicine has performed the project focused on the collaboration amongst PPPM-related professional groups and policymakers. This project was aimed at identifying problems and deficits in healthcare of common and pandemic chronic diseases. Interviews performed with the experts from all countries of the European Union, Israel, Russia and Turkey resulted in the conclusion that one of the common deficits is a missing communication and collaboration between individual professional groups on one side and healthcare professionals and decision-makers on the other side. Moreover, there are difficulties in accessing national and EU regulatory bodies to enhance the message of patient-focused healthcare.

For the paradigm shift from delayed reactive to predictive, preventive and personalised medicine, a new culture should be created in communication between individual professional domains, between doctor and patient, as well as in communication with individual social (sub)groups and patient cohorts. Considering this long-term mission, both societies EPMA and IFCARS have created the common scientific and communication platform which successfully works since 2009 [2-7]. This platform might be considered as the proof of principle in multi-disciplinary collaboration and trans-domain education in PPPM.

Further, to cover currently persisting deficits, EPMA/IFCARS experts will create a permanent information stream targeted at the regulating and standardising bodies to promote national and European programmes and guidelines for PPPM-related research and implementation.

## Model-guided medicine as the essential instrumental part of PPPM

In the last half century, we have seen astonishing strides in medicine generally and in radiology and surgery specifically. Many of these technical advances have found their way into the frontlines of health care. Life-saving, diagnostic and therapeutic technologies and procedures, that would have been unimaginable a few short years ago, have become commonplace in today's modern hospitals. For example, advances in diagnostic medical imaging, in coronary artery interventions, in orthopaedic joint replacements, in chemotherapy and radiation therapy for oncology patients, just to name a few, have improved quality of life and lengthened life for millions of people around the world. These advances have been made possible due to human ingenuity applied in fields such as the basic health sciences, engineering, mathematics and computer sciences.

These same scientific advances have, in part, resulted in an explosion of medical information and of patient-related images, vital signs and other data, which are used by physicians in determining diagnosis, prognosis and treatment plans for patients. To the practicing physician, this has been a double-edged sword. Wherever possible, patients are diagnosed and treated according to medical guidelines based on the general knowledge derived from observations of large populations of patients and controls, i.e., evidence-based medicine (EBM).

However, the use of modern information communication technology (ICT) in clinical decision support has not kept pace with the rate of increase of the methods and technologies for other medical activities. For example, it is no longer possible for a busy physician to keep up with all of the new developments in medical science and to integrate this information with vast quantities of individual patient data for transparent and reproducible decision making. And, even with all of this information, there are still wide variations between individual patients, in terms of anatomy, physiology, metabolism and genetics, that cannot be accounted for or factored into medical procedure decisions by EBM or by standard methodologies of patient assessment that are currently available to physicians and the healthcare systems. This poses the challenge to transfer medical practice from the traditional medical record centric view of the patient towards an ICT-based holistic presentation of the individual patient and corresponding medical procedures.

## Understanding and modelling the patient situation

In real-life clinical settings, a combination of all available medical information, obtained from the medical record and elsewhere, has to be mentally integrated by the physician to create an abstract ‘model’ of the patient, which must be as close as possible to reality to serve as a basis for decision making with respect to the medical procedure to be followed. This is commonly known as clinical judgment.

**Figure 1:**
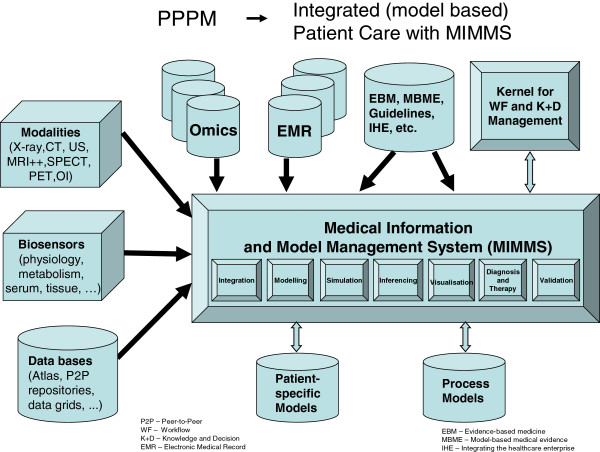
ICT component architecture for integrated (model-based) patient care with MIMMS.

Transcending this traditional medical record centric activity, i.e. information component listing and summary report of the patient/human, we propose a strategy towards an ICT-based holistic presentation of the individual patient and corresponding medical process/procedure redesigned within a given domain of discourse, such as cardiovascular, neurological, diabetic or oncologic disorders; see Figure 1.

ICT systems support provided by a Medical Information and Model Management System (MIMMS)-like architecture with the core functionalities as indicated in Figure 1 is the prerequisite for an effective PPPM. The development and exploration of these functionalities can only be accomplished in a synergistic spirit with disciplines such as medicine, informatics, engineering and mathematical modelling as well as with industry and other stakeholders.

With a holistic presentation of a specific patient, based on appropriate mathematical modelling methods, such as probabilistic relational models and process models as well as advanced ICT-enabling tools, the practice of medicine will be substantially transformed towards model-based medical evidence (MBME) providing transparency of clinical situations, processes and decisions for patient and physician.

To assist physicians in dealing with the overwhelming amount of information that is available, appropriate clinical support tools as part of an advanced ICT MIMMS allow the physician to prioritise and integrate all relevant individual patient data into a formal ICT-based patient-specific model (PSM) to assist in the decision-making processes. Such a model would represent all of the health and disease aspects of a patient, and when linked to medical process models, will lead to a more accurate methodology for a new personalised, patient-specific diagnosis, prognosis and treatment paradigm: model-guided medicine (MGM).

## Medical process/workflow modelling

Organised activities such as those observed in medical diagnostic and therapeutic procedures, regardless of complexity, may be better understood and characterised through workflow modelling, representation and analysis. By analysing, synthesising and filtering multi-component medical processes into their fundamental functional components, a workflow diagrammatical representation as well as appropriate mathematical models may be generated. To provide consistency and reproducibility, these representations must utilise a uniform and consistent ontology. The process models (PM) thus generated may be viewed at different levels of granularity or orders. These may be described from the broadest categories (first-order processes) through the finest levels of the medical procedure (*n*-order process).

The specific process models generated through precise and analytic description of actual medical procedures may be further distilled into generic or reference procedure models for categories of procedures. The reference PMs thus generated provide the underlying roadmap to be followed by a Medical Information and Model Management System throughout an entire medical procedure.

In summary, it can be stated that the real-time linkage between the PSM and the PM is a major feature of a model-guided medicine and represents one of the great challenges of the paradigm of integrated healthcare.

## Information and communication technology for a model-guided medicine

ICT applied in a synergistic spirit with disciplines such as biology, medicine, engineering and mathematical modelling (i.e. a comprehensive presentation of patient-specific situations and medical processes) as well as with industry and other stakeholders is the prerequisite for the fundamental change of medicine as discussed above.

**Figure 2:**
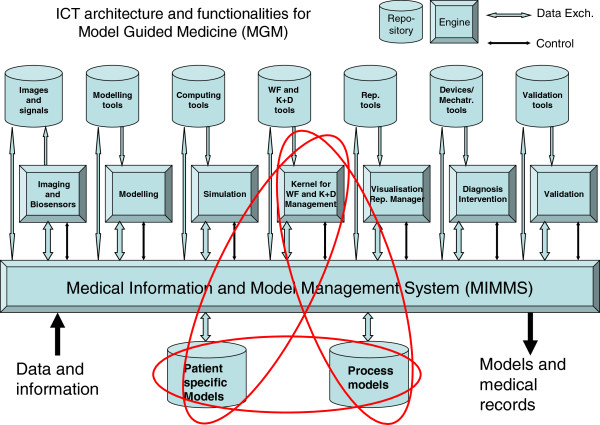
ICT functional architecture for model-guided medicine (MGM) (the ellipsoids include the core competences for a PPPM).

To assist physicians in dealing with the overwhelming amount of information that is available, appropriate clinical support tools as part of an advanced ICT Medical Information and Model Management System, see Figure 2, need to be developed.

The tools provided as part of the MIMMS-type infrastructure allow the physician to prioritise and integrate all relevant individual patient data into a formal ICT-based individual patient profile represented as a patient-specific model. A PSM represents the relevant health and disease aspects of a patient and will help lead to a more accurate methodology for a new personalised, patient-specific diagnosis, prognosis and treatment paradigm, i.e. a model-guided medicine. ICT tools of MIMMS, such as intelligent agents as part of the Kernel for WF and K + D Management, assist in the decision-making processes for a PPPM.

**Figure 3:**
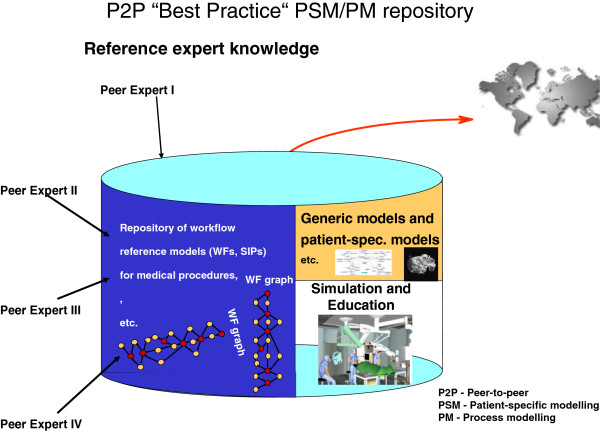
P2P ‘best practice’ PSM/PM representations for PPPM.

The trans-domain integration of expertise of selected specialities will lead to the creation of a PPPM database. This database supports a P2P cooperative environment (see Figure 3) for creating and sharing PSMs and PMs for selected clinical domains and can be extended to additional clinical domains as experience is gained in the networking environment.

Once the information and communication technology is developed, its dissemination throughout all parts of the world, wherever IT and communication interfaces are available, will result in profound and cost-effective modernisation of healthcare. The beneficiaries of these transforming methods and technologies will include patients, healthcare providers and society at large.

## Healthcare impacts expected within the model-guided medicine

It is claimed in this outline on model-guided medicine that the effective use by physicians and patients of the profound advances in medical knowledge and technology in recent decades will only be possible when a new systematised approach to patient and medical procedure modelling and management, utilising advanced ICT methods and tools as described in this proposal, has been developed. This new systematised approach is necessary as off-the-shelf generic forms of decision support software, database management and data mining cannot be simply applied to medical reasoning and knowledge, due to the complexities of human physiology, pathology and, in a healthcare setting, to the corresponding medical procedures.

We are proposing a comprehensive, multi-component model-guided medicine system, based on the visionary concept of a Medical Information and Model Management System that has the ability to achieve the desired results and can be built upon advanced ICT technology in a cost-effective manner and within a reasonable time frame. The key features of this proposed system concept (1) have been conceptualised with input from a wide community of medical practitioners, (2) have been presented and discussed at international medical meetings and associated forums, (3) have been peer reviewed and published, (4) have been and are supported by a federal and local state government for exploratory and highly innovative projects, (5) have received expression of interest by leading industry in the medical field and (6) are supported by an international team whose expertise covers all of the component disciplines, including the design and management of successful large-scale ICT projects for healthcare settings.

In this context, MGM based on the concepts of a PSM, PM and MIMMS will be a forerunner to mainstream research and development and thereby will provide innovative European industry (small, medium and large) new opportunities for providing groundbreaking products and services in healthcare settings.

ICT applied in a synergistic spirit with disciplines such as biology, medicine, engineering, informatics and mathematical modelling (i.e. a comprehensive presentation of patient specific situations and medical processes) as well as with industry and other stakeholders is the prerequisite for the fundamental change of medicine as discussed above. This is accomplished by close cooperation with expert partners from EPMA, IFCARS and other groupings of excellence in healthcare, medicine, education and ICT. After successful realisation of the ICT MIMMS concept, the reproducibility, effectiveness, economics and patient outcome of medical procedure will experience a dramatic improvement.

In the long term, the groundbreaking model-guided medicine vision and system that we are proposing, if designed, implemented and employed as we suggest, will be disruptive to current medical practice and has the potential to transform medical practice into a rigorously scientific-based activity. Physicians and patients will be able to use and leverage the vast amounts of medical information and knowledge from medical disciplines, to have the tools to provide for cost-effective and improved health care and to develop a new means of collecting statistically valid medical data from a model-based medical evidence. We also believe that once the information and communication technology is developed, employed and assessed will substantially transform medical education and training. With this essential educational component, the dissemination of model-guided medicine and associated technologies throughout all parts of the world, wherever IT and communication interfaces are available, will result in profound and cost-effective modernisation of healthcare. The beneficiaries of these transforming methods and technologies will include patients, healthcare providers and society at large.

## Opportunities to realised long-term strategies within ‘Horizon 2020’

**Figure 4:**
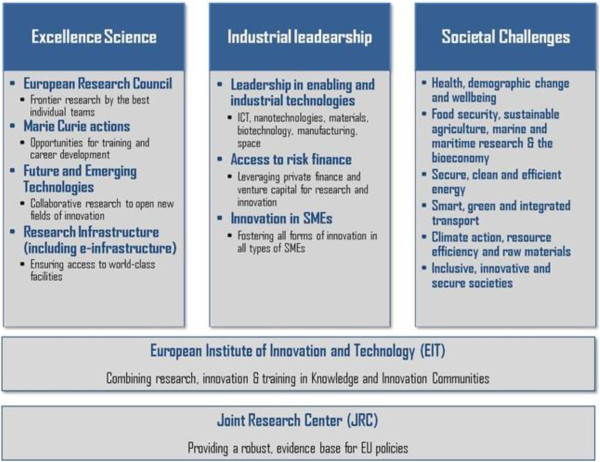
**These priorities correspond to those of Europe 2020 and the Innovation Union, taken from **[1]**.**

The new European programme ‘Horizon 2020’ is, in general, focused on three distinct yet mutually reinforcing priorities, namely, excellent science, industrial leadership and societal challenges (see Figure 4) [1].

### Excellent science

This will raise the level of excellence in Europe's science base and ensure a steady stream of world-class research to secure Europe's long-term competitiveness. It will support the best ideas, develop talent within Europe, provide researchers with access to priority research infrastructure and make Europe an attractive location for the world's best researchers.

This will

● support the most talented and creative individuals and their teams to carry out frontier research of the highest quality by building on the success of the *European Research Council*;

● fund collaborative research to open up new and promising fields of research and innovation through support for *Future and Emerging Technologies* (FET);

● provide researchers with excellent training and career development opportunities through the *Marie Skłodowska-Curie Actions* (*'Marie Curie Actions'*);

● ensure Europe has world-class *research infrastructures* (including e-infrastructures) accessible to all researchers in Europe and beyond.

### Industrial leadership

This will aim at making Europe a more attractive location to invest in research and innovation (including eco-innovation), by promoting activities where businesses set the agenda. It will provide major investment in key industrial technologies, maximise the growth potential of European companies by providing them with adequate levels of finance and help innovative SMEs to grow into world-leading companies.

This will

● build *leadership in enabling and industrial technologies*, with dedicated support for ICT, nanotechnologies, advanced materials, biotechnology, advanced manufacturing and processing, and space, while also providing support for cross-cutting actions to capture the accumulated benefits from combining several key enabling technologies;

● facilitate *access to risk finance*;

● provide Union-wide support for *innovation in SMEs*.

### Societal challenges

This reflects the policy priorities of the Europe 2020 strategy and addresses major concerns shared by citizens in Europe and elsewhere. A challenge-based approach will bring together resources and knowledge across different fields, technologies and disciplines, including social sciences and the humanities. This will cover activities from research to market with a new focus on innovation-related activities, such as piloting, demonstration, test beds, and support for public procurement and market uptake. It will include establishing links with the activities of the European Innovation Partnerships.

Funding will be focussed on the following challenges:

– *Health, demographic change and wellbeing;*

– *Food security, sustainable agriculture, marine and maritime research and the bio-economy;*

– *Secure, clean and efficient energy;*

– *Smart, green and integrated transport;*

– *Climate action, resource efficiency and raw materials;*

– *Inclusive, innovative and secure societies.*

The EIT will play an important role by combining excellent research, education and innovation, thus integrating the knowledge triangle. The EIT will do so primarily through the Knowledge and Innovation Communities (KICs). In addition, it will ensure that experiences are shared beyond the KICs through targeted dissemination and knowledge-sharing measures.

The Joint Research Centre's activities will be an integral part of Horizon 2020, providing robust, evidence-based support to Union policies. This will be driven by customer needs complemented by forward-looking activities.

Horizon 2020 will be a 7-year programme, and there may be significant shifts in the broader economic and policy context as the programme progresses. Ensuring Horizon 2020's continued relevance will therefore also require adjustment of priorities and resources, as and when necessary. As such, flexibility clauses have been included in the proposal in this respect.

The implementation of Horizon 2020 will also take a strategic approach to programming of research and innovation, using joint actions and modes of governance aligning closely with policy development yet cutting across the boundaries of traditional sectoral policies. This will be based on sound evidence, analysis and foresight, with progress measured against a robust set of indicators.

As regards the funding of research activities involving human embryonic stem cells, the Horizon 2020 legislative package is fully in line with the approach supported by the European Parliament and the Council upon their adoption of the FP7 legislation, as set out in the Commission's statement of 2006 (OJ L412 of 30 December 2006).

## Specific calls (PHC) 2014–2015 dedicated to the issues of the model-guided medicine

The work programme for 2014–2015 [8] consists of the altogether seven thematic areas with 34 scientific topics for project proposals, 10 co-ordination actions, and other activities such as SME promotion. Thereby, several calls listed below may be considered for the projects directly or indirectly dedicated to the effective promotion of the field of model-guided medicine.

1. Understanding health, ageing and disease

● PHC 2 – 2015: Understanding diseases: systems medicine

● PHC 3 – 2015: Understanding common mechanisms of diseases and their relevance in co-morbidities

2. Effective health promotion, disease prevention, preparedness and screening

● PHC 4 – 2015: Health promotion and disease prevention: improved inter-sector co-operation for environment and health based interventions

3. Improving diagnosis

● PHC 10 – 2014: Development of new diagnostic tools and technologies: *in vitro* devices, assays and platforms

● PHC 11 – 2015: Development of new diagnostic tools and technologies: *in vivo* medical imaging technologies

● PHC 12 – 2014/2015: Clinical research for the validation of biomarkers and/or diagnostic medical devices

4. Innovative treatments and technologies

● PHC 13 – 2014: New therapies for chronic non-communicable diseases

● PHC 16 – 2015: Tools and technologies for advanced therapies

5. Advancing active and healthy ageing

● PHC 19 – 2014: Advancing active and healthy ageing with ICT: service robotics within assisted living environments

● PHC 20 – 2014: Advancing active and healthy ageing with ICT: ICT solutions for independent living with cognitive impairment

● PHC 21 – 2015: Advancing active and healthy ageing with ICT: early risk detection and intervention

6. Integrated, sustainable, citizen-centred care

● PHC 23 – 2014: Developing and comparing new models for safe and efficient, prevention-oriented health and care systems

● PHC 24 – 2015: Piloting personalised medicine in health and care systems

● PHC 25 – 2015: Advanced ICT systems and services for integrated care

● PHC 26 – 2014: Self-management of health and disease: citizen engagement and mHealth

● PHC 27 – 2015: Self-management of health and disease and patient empowerment supported by ICT

● PHC 28 – 2015: Self-management of health and disease and decision support systems based on predictive computer modelling used by the patient him or herself

● PHC 29 – 2015: Public procurement of innovative eHealth services

7. Improving health information, data exploitation and providing an evidence base for health policies and regulation

● PHC 30 – 2015: Digital representation of health data to improve disease diagnosis and treatment

● PHC 31 – 2014: Foresight for health policy development and regulation

● PHC 32 – 2014: Advancing bioinformatics to meet biomedical and clinical needs

● PHC 33 – 2015: New approaches to improve predictive human safety testing

● PHC 34 – 2014: eHealth interoperability

More detailed information about the ‘Horizon 2020’ and specific calls can be found in the ‘EPMA Position Paper’ [1].

## Expert recommendations

Consolidation of professional groups involved in PPPM-related research and healthcare services is a multifunctional task which should be performed at several levels of activities:

– Mandated optimal set-up of multidisciplinary stakeholders in diagnostics and in the prevention and treatment of individual pathologies

– Regular meetings of stakeholders with multidisciplinary expertise motivated and regulated by healthcare-related national and international programmes

– Multidisciplinary education as an obligation for related professional groups

– Creation of optimal economic conditions motivating a multidisciplinary expertise to be applied in healthcare sciences and services

– Creation of international multidisciplinary projects within ‘Horizon 2020’ and alternative programmes

– Interdisciplinary analysis of the projects submitted and approved within ‘Horizon 2020’ and alternative programmes: output, impacts, sustainability and recommendations

– Creation of new guidelines for PPPM-related application in healthcare.

Both societies EPMA and IFCARS wish to promote the idea of the dialogue with other consolidated professional groups who may consider common long-term strategies in PPPM and consequent projects within ‘Horizon 2020’ and alternative programmes.

## Competing interests

The authors declare that they have no competing interests.

## Authors' contributions

HL and OG have drafted the manuscript and circulated it with the members of both EPMA and IFCARS in order to finalise the position paper. Both authors read and approved the final manuscript.

## Author's information

The article is published on behalf of the European Association for Predictive, Preventive and Personalised Medicine and International Foundation for Computer Assisted Radiology and Surgery and expresses the consolidated position of their members and representatives.
